# Topological Quantum Phase Transition and Local Topological Order in a Strongly Interacting Light-Matter System

**DOI:** 10.1038/s41598-017-01726-z

**Published:** 2017-05-12

**Authors:** Sujit Sarkar

**Affiliations:** Poornaprajna Institute of Scientific Research, 4 Sadashivanagar, Bangalore, 5600 80 India

## Abstract

An attempt is made to understand the topological quantum phase transition, emergence of relativistic modes and local topological order of light in a strongly interacting light-matter system. We study this system, in a one dimensional array of nonlinear cavities. Topological quantum phase transition occurs with massless excitation only for the finite detuning process. We present a few results based on the exact analytical calculations along with the physical explanations. We observe the emergence of massive Majorana fermion mode at the topological state, massless Majorana-Weyl fermion mode during the topological quantum phase transition and Dirac fermion mode for the non-topological state. Finally, we study the quantized Berry phase (topological order) and its connection to the topological number (winding number).

## Introduction

To get a better understanding of quantum many-body systems, which show topological properties, one has to focus on the studies of non-local order parameter instead of conventional concept of symmetry breaking, i.e., the local order parameter description of the system. States with non-local order parameter could be called topologically ordered. The term “topological” implies the existence of a bulk invariant, which generally represented by an integer that differentiates between the phases of matter having the same symmetry^1, 2^. The generalized concept of topological numbers such as Chern number and winding number depends on the dimensionality of the system for a bulk characterization based on the concept of topological order^1, 2^.

The concept and existence of Majorana fermion mode is one of the most advanced research area in the quantum condensed matter physics for studying the topological properties of matter. Majorana introduced a special kind of fermions which are their own antiparticles^3–5^. In the last decade, physics of topological state of matter through the analysis of Majorana mode, come to the focus of quantum many body condensed matter physics research^6–29^. The physics of p-wave pairing in a condensed matter many body system has been considered as the prototype system for investigating the physics of Majorana fermion zero mode which is localized at the both ends of the system^1^.

In the strongly correlated regime of interacting light matter physics, it is possible to generate an effective strong repulsion between the photons, and from this study, one can understand and quantum simulate different interesting physical properties of strongly correlated quantum condensed matter many body system.

In quantum simulation, one’s aim is to simulate a quantum system using a controllable laboratory system that underlines the same mathematical models. Therefore, it is possible to simulate a quantum system that can neither be efficiently simulated on a classical system nor easily be accessed experimentally^30–32^.

Interacting light-matter system is also a very good platform to simulate different quantum many-body physics phenomena. Several interesting phenomena like fermionic behavior of photons, driven dissipative Mott insulator, Tonk-Girardeau gas, fractional quantum Hall like state and the physics of cavity QED lattice and quantum simulation of many complicated quantum many-body system have found place in the literature of interacting light-matter physics^30–39^.

The recent experimental success in engineering, a strong interaction between the photons and the atoms in high quality micro-cavities opens up the possibility to use the light matter system as quantum simulators for many body physics. Many interesting results are coming out to understand the complicated quantum many-body system^34–39^.

The authors of ref. 40 have proposed a model for a one dimensional array of nonlinear cavities where they have quantum simulated p-wave pairing effectively arising from the interplay between the strong on-site interaction and two-photon parametric driving. Therefore, this model may have ingredient for the existence of topological properties. The authors have tried to quantum simulate the Majorana like modes for this one-dimensional array of nonlinear cavities, but the detail study of the topological properties and emergence of different relativistic modes have not addressed there. The authors of ref. 41 have studied the non-equilibrium steady state of the driven dissipative version of this model^40^. We will also use the same model Hamiltonian for the present study but in a different context.

## Motivations of this study

The present study has three motivations.

### First motivation

Here we will do the detail study of the topological state and also topological quantum phase transition of the interacting light-matter physics in an array of nonlinear cavities through the topological invariant number. This motivation arises for the following reasons.

Topological quantum phase transition describes with the topologically invariant number while the quantum phase transition describes by order parameter^42, 43^. Topological number changes by an integer number during the topological quantum phase transition from topological state to the non-topological state, which is related to the appearance of Majorana zero modes localized at the edge of the system^1, 5^. This detailed study based on exact calculations too is absent in the previous literature of light-matter physics system^30–41^.

### Second motivation

We would like to find out the emergence of different relativistic modes (massive Majorana fermion mode, massless Majorana-Weyl fermion mode and massless Dirac fermion mode), for this interacting light-matter physics in a nonlinear cavities array, based on the exact solutions. This motivation arises due to the following reasons:

The fundamental constituent of condensed matter quantum many-body system is invariably the electron which carries charge and it has antiparticle. Therefore, to find the Majorana particle in this system, it will appear as a emergent particle. We will prove the emergence of Majorana and Majorana-Weyl collective modes in this strongly correlated light-matter system in a nonlinear cavity array.

To the best of our knowledge, this is the first study in the entire literature^30–41^ of interacting light-matter system to find the emergence of different relativistic modes.

### Third motivation

Here, we find an equivalence between the topological invariant number and the local topological order for this system. This motivation arises due to the following reasons:

The author of ref. 44 has used the concept of the quantized Berry phase to define a local topological order parameter for gapped quantum liquid system which do not require any translation symmetry. The author has assumed that the Hamiltonian has anti-unitary symmetry. The ground state is gapped and unique. The Hamiltonian of the present problem fulfils all the criteria to study the topological properties of the system in terms of quantized Berry phase.

But in the literature of interacting light-matter system, there are no studies so far of quantized geometric phase as a local topological order for characterizing the topological state of the system^30–41^ and its relation to the topological number.

## Model Hamiltonian and basic physical aspects

The model Hamiltonian allows for tunable coupling and nonlinearity. This system consist of N optical cavities coupled through nearest-neighbor (NN) photon tunneling across the one dimensional chain. In this system, each cavity exhibits a large optical nonlinearity and a single mode which behave as a Wannier function localized at the center of each site. Photon tunneling occurs due to the finite spatial overlap between NN Wannier modes. Finally, the system Hamiltonian takes the following form^40^:

1 $${H}_{0}={\omega }_{c}\sum _{i}^{N}{b}_{i}^{\dagger }{b}_{i}+\frac{U}{2}\sum _{i}^{N}{b}_{i}^{\dagger }{b}_{i}^{\dagger }{b}_{i}{b}_{i}-J\sum _{i}^{N-1}({b}_{i}^{\dagger }{b}_{i+1}+h\mathrm{.}c),$$

where ${b}_{i}({b}_{i}^{\dagger })$ are annihilation (creation) operators associated with the i-th cavity of the chain with cavity frequency *ω*_*c*_, *U* is the strength of the on-site photon-photon repulsion due to the large optical nonlinearities and *J* denotes the photon tunneling amplitude of photon between NN site. The photon tunneling occurs owing to the non-vanishing overlap of Wannier modes between the NN sites. In the strong interaction regime, the energy cost for adding any extra photon is higher than all relevant energy scale in the system. Therefore, in each site of the lattice, the number of photon is either 0 or 1, i.e., the photon shows the spinless fermionic behavior.

The authors of ref. 40 have mapped such a system to a spin model. They have proposed the configuration which allows the interactions between the spins and the transverse field. One of the most important ingredients of this model Hamiltonian is the emergence of p-wave pairing and optical version of the Kitaev chain, which we will discuss in the next sections.

### The emergence of effective p-wave pairing for this system

The detail derivation of the emergence of effective p-wave pairing has presented in ref. 40. Here, we mention it very briefly for the completeness of the study. The authors of ref. 40 have introduced parametric pumps which inject pairs of photons into the system through nonlinear optical process where these pumps drive the system locally through the inter-cavity field. This cavity field, consists of a superposition of two neighboring Wannier modes.

In the strongly correlated regime, the photons are likely to be emitted from the NN cavity. The effective drive Hamiltonian^40^ is

2 $${H}_{drive}=-\,|{\rm{\Delta }}|\sum _{i=1}^{N-1}({e}^{(i2{\omega }_{p}t+\varphi )}{b}_{i}{b}_{i+1}+h\mathrm{.}c),$$

where ${\rm{\Delta }}=|{\rm{\Delta }}|{e}^{i\varphi }$, where Δ and *ϕ* are the amplitude and phase of the parametric pump and *ω*_*p*_ is the frequency. Physically, the above Hamiltonian describes the coherent exchange of p-wave paired photons between the system and pump field. The effective p-wave pairing arises from the interplay between the parametric pumping and strong on-site photon-photon repulsion. In the strong interaction regime, where the parametric pumps are much weaker than the on-site photon-photon repulsion, the second process is strongly favored and the p-wave is effectively obtained. The authors have determined the amplitude of |Δ| by the overlap of the Wannier modes in a similar way as the tunneling amplitude, *J* ^40^. Therefore, one can expect that one reaches a regime where the magnitude of |Δ| and *J* are of the same order. We will explore this limit explicitly in the study of topological quantum phase transition and also to find the exact solution for winding number.

### Optical version of Kitaev chain

Here we derive the optical version of Kitaev’s chain starting from the Hamiltonian *H*(=*H*_0_ + *H*_*drive*_) for the strongly correlated regime of the system $(U\gg J,|{\rm{\Delta }}|)$.

In the hard core photon limit, one can write the photonic operators as ${\tilde{b}}_{i}=P{b}_{i}P$ and ${\tilde{b}}_{i}^{\dagger }={({\tilde{b}}_{i})}^{\dagger }$, where *P* is the projection operator for the single photon occupancy. For this case the drive Hamiltonian reduce to

$${H}_{drive}=-\,|{\rm{\Delta }}|\sum _{i=1}^{N-1}({e}^{(i2{\omega }_{p}t+\varphi )}{\tilde{b}}_{i}{\tilde{b}}_{i+1}+h\mathrm{.}c).$$

One can consider this hard-core photon as spin-1/2 particles and express the photon operators as a Pauli matrix, ${\sigma }_{i}^{-}=2{\tilde{b}}_{i}$ and ${\sigma }_{i}^{\dagger }={({\sigma }_{i}^{-})}^{\dagger }$ and their fermionic nature can be unveiled by mapping the spin-1/2 particle to the spinless fermions through the Jordan-Wigner transformations. The analytical relation between the spinless fermion operator and Pauli spin matrices in Jordan-Wigner transformation is ${a}_{i}=(\frac{1}{2}){{\rm{\Pi }}}_{j=1}^{i-1}\,(\,-\,{\sigma }_{j}^{z}){{\sigma }_{i}}^{-}$. One can omit the explicit time dependence by a transformation to a rotating frame of frequency *ω*_*p*_^40, 41^. The total Hamiltonian, *H* = *H*_0_ + *H*_*drive*_, reduce to following form:

3 $${H}_{1}=-\,J\sum _{i=1}^{N-1}({a}_{i}^{\dagger }{a}_{i}+h\mathrm{.}c)+\sum _{i=1}^{N-1}(|{\rm{\Delta }}|{e}^{i\varphi }{a}_{i}{a}_{i+1}+h\mathrm{.}c)-\mu \sum _{i=1}^{N}{a}_{i}^{\dagger }{a}_{i}.$$

This is the optical version of the Kitaev’s chain for this model Hamiltonian. Here, ${a}_{i}({a}_{i}^{\dagger })$ is the annihilation(creation) spinless fermion operators, which represent the physics of fermionized photon. Here, cavity frequency, *ω*_*c*_ playing the role of Fermi energy and the detuning plays the role of chemical potential (*μ* = *ω*_*p*_ − *ω*_*c*_, where *ω*_*p*_ is the frequency of external two photon drive). We show explicitly that the phase of this model Hamiltonian has no effect on the topological properties, we mention it explicitly in the “Method” section. In the next section, we derive the topological invariant number based on this optical Kitaev’s Hamiltonian (*H*_1_), where there is no further $\varphi$ term in *H*_1_.

## Results

### Topological number: A winding number study

Here, we explicitly show that the topological quantum phase transition of the system occurs through a change of topological invariant quantity, i.e., the winding number.

One can write the model Hamiltonian *H*_1_ (Eq. 3), in the momentum space, in the following form

4 $$\begin{array}{rcl}{H}_{1} & = & \frac{1}{2}\sum _{k}{\psi }_{k}^{\dagger }h(k){\psi }_{k}.\\ {h}_{k} & = & (\begin{array}{cc}\epsilon (k) & i{\rm{\Delta }}(k)\\ -i{\rm{\Delta }}(k) & -\epsilon (k)\end{array}),\end{array}$$

where ${\epsilon }_{k}=-\,2J\,\cos \,k-\mu$, ${\rm{\Delta }}(k)=2|{\rm{\Delta }}|\,\sin \,k$. ${\psi }_{k}={({a}_{k},{a}_{-k}^{\dagger })}^{T}$. This Hamiltonian satisfies the condition of anti-unitary particle-hole symmetry, $Ch(k){C}^{-1}=-\,h(k)$, where *C* is the anti-unitary operator. One can also write ${\sigma }_{x}Kh(k)K{\sigma }_{x}=-\,h(\,-\,k)$, where *K* is the complex conjugation operator^17^. This model Hamiltonian has also another symmetry ${\sigma }_{x}h(k){\sigma }_{x}=h(\,-\,k)$. This model Hamiltonian is the one dimensional, *Z* type topological, BdG system. The Bogoliubov quasi-particle operator diagonalize the Hamiltonian. Finally, the Hamiltonian reduces to $H(k)={\sum }_{k}\,{E}_{k}{\beta }_{k}^{\dagger }{\beta }_{k}$, where *β*_*k*_ is the Bogoliubov quasi-particle operator. The detail presentation of this quasi-particle operator and the excitation spectrum are relegated to the “Method” section.

One can also write the Hamiltonian as,

5 $$h(k)=\vec{\chi }(k\mathrm{).}\vec{\tau },$$

where $\vec{\tau }$ are Pauli matrices which act in the particle-hole basis, and ${\chi }_{x}(k)=0$, ${\chi }_{y}(k)=2{\rm{\Delta }}\,\sin \,k$ and ${\chi }_{z}(k)=-\,2J\,\cos \,k-\mu$. It is convenient to define this topological invariant quantity using the Anderson pseudo-spin approach^45^.

6 $$\vec{\chi }(k)={\rm{\Delta }}(k)\vec{y}+({\epsilon }_{k}-\mu )\,\vec{z}\mathrm{.}$$

It is very clear from the analytical expression that the pseudo spin defined in the *y* − *z* plane,

7 $$\hat{\chi }(k)=\frac{\vec{\chi }(k)}{|\overrightarrow{\chi }(k)|}=\,\cos \,({\theta }_{k})\,\hat{y}+\,\sin \,({\theta }_{k})\,\hat{z}\mathrm{.}$$

8 $${\theta }_{k}={\rm{t}}{\rm{a}}{{\rm{n}}}^{-1}\,(\,-\,(2J\,\cos \,k+\mu \mathrm{))/(2}{\rm{\Delta }}\,\sin \,k).$$

Here the momentum states with periodic boundary condition for a ring *T* ^(1)^ and the unit value $\hat{\chi }(k)$ exists on a unit circle *S* ^(1)^ in the *y*–*z* plane. Therefore, *θ*(*k*) is a mapping. ${S}^{\mathrm{(1)}}\Rightarrow {T}^{\mathrm{(1})}$ and the topological invariant is simply the fundamental group of the mapping which is just the integer winding number. It is only an integer number and,therefore, can not vary with smooth deformation of the Hamiltonian as long as the quasi-particle gap remains finite. At the point of topological phase transition the winding number changes discontinuously.

The analytical expression for winding number (*W*) is

9 $$W=(\frac{1}{2\pi }){\int }_{-\pi }^{\pi }(\frac{d{\theta }_{k}}{dk})dk=(\frac{1}{2\pi }){\int }_{-\pi }^{\pi }\frac{2{\rm{\Delta }}\mathrm{(2}J+\mu \,\cos \,k)}{{(\mu +2J\cos k)}^{2}+4{{\rm{\Delta }}}^{2}\,{\sin }^{2}\,k}dk.$$

This winding number describes the total number of that unit vector $\overrightarrow{\chi }(k)$ travels counter-clockwise around the origin in the *y*–*z* plane. There are some other representation^29^ for the calculation of winding number but that too finally gives the same result as the Anderson pseudo-spin approach^45^. We will present the other representation and the equivalence between them in the “Method” section.

### Topological quantum phase transition

The topological quantum phase transition is characterized by the following observations: A discontinuity in the topological number (winding number) and massless excitation at the point of topological quantum phase transition, which implies that system has diverging length scale. We calculate the topological number based on the study of winding number (Eq. 9).

**Figure 1:**
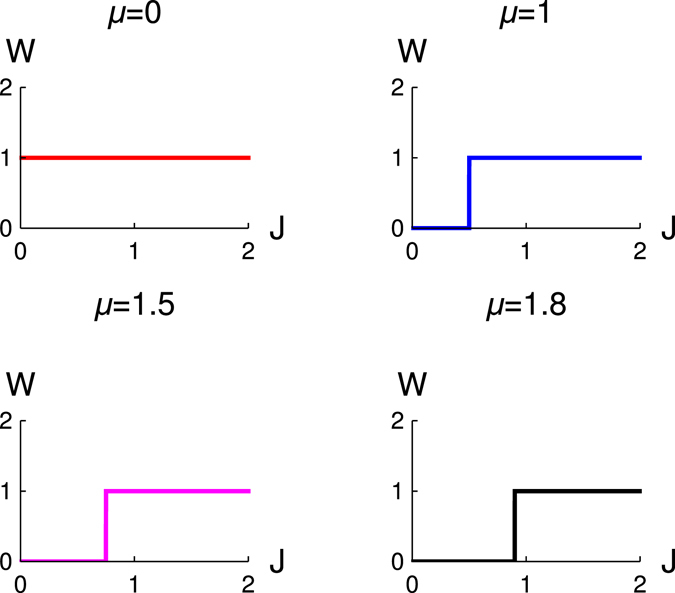
The variation of winding number (*W*) with *J* for different values of *μ*. Different figures are for different values of *μ*, red (*μ* = 0), blue (*μ* = 1.0), magenta (*μ* = 1.5) and black (*μ* = 1.8), Δ = 1.

In Fig. 1, we present the results of the variation of winding number (*W*) with *J*. It is clear from our study that there is topological quantum phase transition from non-topological quantum state (*W* = 0) to topological state (*W* = 1). We study for different values of chemical potentials, and we observe that topological quantum phase transition occurs at *J* = *μ*/2 for a fixed value of Δ (here Δ = 1). But at *μ* = 0, the system is always in the topological state, and there is no topological quantum phase transition. We also observe that the change of the winding number is unity during the topological quantum phase transition and that there is no further change of winding number. For such a situation only one Majorana zero mode appears at both the ends of this optical Kitaev chain. In the previous study^40^, the authors of this proposed model only predict appearance of Majorana fermions at the edge of the chain for Δ = *J* > 0 and *μ* = 0.

**Figure 2:**
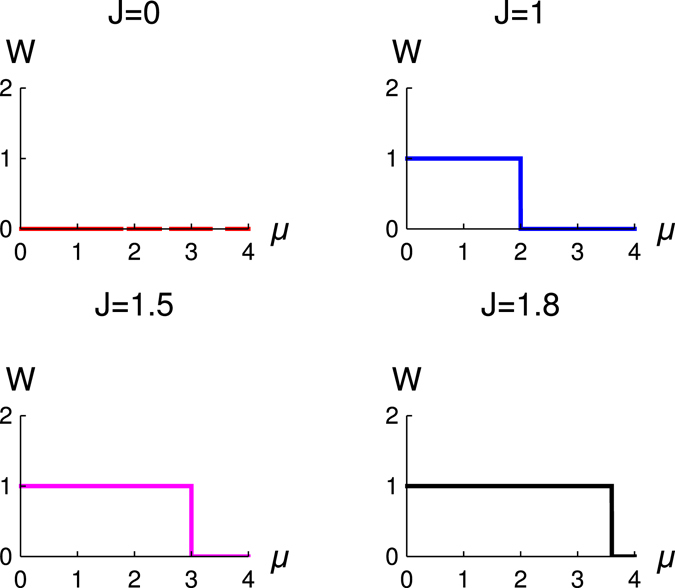
The variation of winding number (*W*) with *μ* for different values of *J*. Different figures are for the different values of *J*, red (*J* = 0.5), blue (*J* = 1), magenta (*J* = 1.5) and black (*J* = 1.8), Δ = 1.

In Fig. 2, we study the variation of *W* with *μ*. These figures are for the different values of *J* but for Δ = 1. We observe that topological quantum phase transition occurs for *μ* = 2*J*. It is also consistent with the result of Fig. 1, which is a check for the consistency of the study of topological number. The behaviour of topological phase transition is the same for narrow and wide band, and it always obey the same relation between the *μ* and *J*, i.e., *μ* = 2*J*.

**Figure 3:**
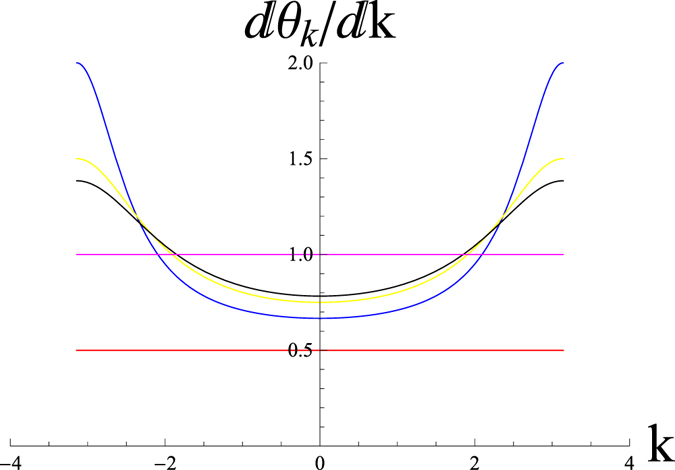
The variation of $\frac{d{\theta }_{k}}{dk}$ with *k*. The curve for the magenta color (*W* = 1) is for *μ* = 0 and the other curves are for *μ* = 1. Here we consider Δ = *J*. Different curves are for the different values of (Δ = *J*). Red (0.5), blue (1), yellow (1.5) and black (1.8).

Figure 3 shows the variation of $\frac{d{\theta }_{k}}{dk}$ with *k* for two values of chemical potentials (*μ* = 0, 1). It is clear from our study for *μ* = 0 (*W* = 1, magenta curve), there is no variation of $\frac{d{\theta }_{k}}{dk}$ with *k* and it merges in a single line with the value unity. Therefore, the system is always in the topological state for equal values of Δ = *J* with no topological quantum phase transition. But for the finite values of the chemical potential ($\mu \ne 0$), $\frac{d{\theta }_{k}}{dk}$, shows the variation with *k*. The behavior for different values of Δ = *J* are different. Therefore for this limit, system is not always in the topological state but it also shows the topological quantum phase transition. For this situation, the pseudo-spin vector rotates once in the *y*–*z* plane around the origin.

We would like to explain this result more physically: The model Hamiltonian parameters Δ and *J* are equal, and also $\mu \ne 0$, i.e., the finite detuning between the cavity frequency and the parametric pumping leads the topological quantum phase transition. But in the resonance condition, i.e., when *ω*_*p*_ = *ω*_*c*_, the system is always in the topological state. This limit of the parameter space is physically feasible as discussed in ref. 40. Therefore depending on the difference of the values of *ω*_*p*_ and *ω*_*c*_, the system is either in a topological state or in a non-topological state. We will also discuss this point again during the study of exact solutions.

The authors of ref. 28 have studied the topological quantum phase transition for an extended Kitaev’s model and Su-Schrieffer and Higger model based on the topological invariant quantity. They have found a few interesting results. But in the present study, we find the topological state and the topological quantum phase transition of the interacting light-matter physics for nonlinear cavity arrays through the study of topological invariant quantity of optical Kitaev’s chain.

Our study and results, present a new and significant advance in the study of topological state and the topological quantum phase transition for strongly interacting light-matter system in a nonlinear cavities array, which is absent in the literature of light-matter physics^30–41^.

### Results based on exact solutions and physical explanation

The analytical expressions of winding number is not solvable exactly for the whole range of parameter space. But one can find the exact solution for a few regime of the parameter space. We use those exact solutions explicitly during the further analysis of this study. From this exact solution, we show that the excitations at the topological quantum phase transition is massless Majorana-Weyl fermion mode and,therefore, the system is in the quantum critical state at this topological quantum phase transition point.

### A few exact calculations based results for winding number study

We find a few exact solutions for our model Hamiltonian in different regime of parameter space and also discuss the related physics.

First, we consider the situation, when Δ = *μ* = *J*. In this case, the analytical expression for topological number become, $W=\frac{1}{\pi }{\int }_{-\pi }^{\pi }\,\frac{2+\,\cos \,k}{5+4\,\cos \,k}dk=1$. For this case, the system is in the topological state and the result is also consistent with the study of Figs 1 and 2, where we predict that topological quantum phase transition occurs when *μ* = 2*J*. Therefore, for *μ* = *J*, the system is in the topological state without any topological quantum phase transition.

Secondly, we consider the case when *μ* = 0, but Δ and *J* are finite. The analytical expression for winding number become $W=\frac{1}{2\pi }{\int }_{-\pi }^{\pi }\,dk$. If Δ = *J* then the winding number become unity. For this case, system is always in the topological state. This exact result is consistent with the study of Fig. 3 for *μ* = 0 curve.

Thirdly, we consider the situation when Δ is very small but finite and *J* is also finite, i.e, $c=\frac{{\rm{\Delta }}}{J}\sim 0$. If we expand the integrand up to the order *c* ^5^ then the expression for winding number is, $W=\frac{1}{2\pi }{\int }_{-\pi }^{\pi }\,[ck+\mathrm{(1/3})(c-{c}^{3}){k}^{3}+\mathrm{(1/15})(2c-5{c}^{3}+3{c}^{5}){k}^{5}]dk$. This integral is odd function of *k* as a result of which the winding number is zero and the system is in the non-topological state. This exact result is also consistent with physically.

Finally, we consider the case when Δ is finite, *μ* = Δ/2 and *J* = Δ/2. The analytical expression for winding number become $W=\frac{1}{\pi }{\int }_{-\pi }^{\pi }\,\frac{1+0.5\,\cos \,k}{{\mathrm{(0.5}+\cos k)}^{2}+4\,{\sin }^{2}\,k}dk=1$. This exact result implies that the system is in the topological state and consistent with the result of Figs 1 and 2. The main message of this exact solution is that the condition for the topological state (*μ* = *J*) remains for any values of Δ s as far as Δ is finite. We use these exact solutions based results in the next section to study the emergence of different relativistic modes of the system.

This exact solution based calculations and results for the topological properties of light are absent in the entire literature of interacting light matter system^30–41^.

### Dirac equation for massive Majorana fermion mode and massless Majorana-Weyl mode

The topological properties of the system motivates us for the searching of different kind of relativistic modes as an emergent property of the Hamiltonian. Here, we present the results and the physical explanation of Dirac equation for Majorana fermion mode for the system. At first we present the effective Hamiltonian of the system in the strongly correlated regime ($U\gg J,{\omega }_{c}$).

We can write the Hamiltonian as

10 $$H=-\,J\sum _{j}({b}_{j}^{\dagger }{b}_{j}+h\mathrm{.}c)+{h}_{j}.$$

The on-site Hamiltonian *h*_*j*_,

11 $${h}_{j}={\omega }_{c}{b}_{j}^{\dagger }{b}_{j}+U{b}_{j}^{\dagger }{b}_{j}^{\dagger }{b}_{j}{b}_{j},$$

where *ω*_*c*_ is the cavity frequency and *U* is the on-site photon-photon repulsion.

As we have already mentioned in the previous paragraphs that near to the resonance condition (*ω*_*p*_ ~ *ω*_*c*_) under the presence of two photon drive Δ cos (2*ω*_*p*_ *t*) and also in large *U*. For this conditions the two photon pump is only resonant for the creation of pairs of photons on NN cavities. In the limit of fermionized photonic mode, one can consider each cavity mode as a spin-1/2 particle as we have done in the previous section during the derivation of Kitaev’s model. Finally following the ref. 41, we write the Hamiltonian in Pauli spin operators as,

12 $${H}_{2}=\frac{{\omega }_{c}}{2}\sum _{j}{\sigma }_{j}^{z}-J\sum _{j}({\sigma }_{j}^{\dagger }{\sigma }_{j+1}^{-}+h\mathrm{.}c)-{\rm{\Delta }}\sum _{j}({\sigma }_{j}^{\dagger }{\sigma }_{j+1}^{\dagger }{e}^{-2i{\omega }_{p}}+h\mathrm{.}c\mathrm{).}$$

We observe the explicit time dependence in the above Hamiltonian. One can omit that explicit time dependence by a transformation to a rotating frame,

13 $${H}_{2}=-\,J\sum _{j}[g{\sigma }_{j}^{z}+({\sigma }_{j}^{\dagger }{\sigma }_{j+1}^{-}+h.c)+(\frac{{\rm{\Delta }}}{J})({\sigma }_{j}^{\dagger }{\sigma }_{j+1}^{\dagger }+h\mathrm{.}c)],$$

where $g=\frac{{\omega }_{p}-{\omega }_{c}}{2J}$. After a few steps of calculations, we can write the above Hamiltonian in the form of Ising model,

14 $${H}_{2}=-\,J\sum _{j}[g{\sigma }_{j}^{z}+\frac{1+(\frac{{\rm{\Delta }}}{J})}{2}{\sigma }_{j}^{x}{\sigma }_{j+1}^{x}+\frac{1-(\frac{{\rm{\Delta }}}{J})}{2}{\sigma }_{j}^{y}{\sigma }_{j+1}^{y}].$$

The above Hamiltonian reduced to the transverse Ising model when Δ = *J*, which is physical limit ref. 40. We obtain from the exact solutions that the system is in the topological state for the situation Δ = *J* and *μ* = 0 and also from the study of winding number that the system is in the topological state as far as *μ* < 2*J* for finite Δ. Therefore, the Hamiltonian *H*_2_ in this limit corresponds to the topological state.

15 $${H}_{2}=-\,J\sum _{j}[g{\sigma }^{x}+{\sigma }_{j}^{z}{\sigma }_{j+1}^{z}]\mathrm{.}$$

Now we would like to quantum simulate Majorana fermion mode for this system through the derivation for Dirac equation of Majorana fermion mode. For that purpose, we introduce the order and disorder operators (please see the “Method” section for explicit relations). These operators are defining the sites of the lattice (we define the operator between the NN site of the original lattice). Our calculations are as exact as possible.

Here, we define the Dirac spinor, ${\chi }_{1}(n)={\sigma }_{z}(n){\mu }_{z}(n+\mathrm{1/2)}$ and ${\chi }_{2}(n)={\sigma }_{z}(n){\mu }_{z}(n-\mathrm{1/2)}$, where *σ*s and *μ*s are the order and disorder respectively. These two fields, ${\chi }_{1}(n)$ and ${\chi }_{2}(n)$ satisfy, the following relations, $\{{\chi }_{1}(n1),{\chi }_{2}(n\mathrm{2)}\}=2{\delta }_{n1,n2}$, and ${\chi }_{1,2}^{\dagger }={\chi }_{1,2}$.

One can write down the final equation in the following form,

16 $$({\gamma }^{0}\frac{\partial }{\partial t}+{\gamma }^{3}\frac{\partial }{\partial r}+m)\chi (x)=0.$$

The detailed derivation is relegated to the “Method” section.

One can also write the above Majorana equation in a compact form:

17 $$(i{\tilde{\gamma }}^{\mu }{\partial }_{\mu }-m)\chi (x)=\mathrm{0,}$$

where $${\tilde{\gamma }}^{0}=(\begin{array}{cc}0 & i\\ i & 0\end{array})$$, $${\tilde{\gamma }}^{3}=(\begin{array}{cc}i & 0\\ 0 & -i\end{array})$$, *m* = 1 − *g*.

Therefore, we prove that the spinor field satisfies the Majorana condition of Majorana fermion mode and also the $\tilde{\gamma }$ matrices are imaginary. The massless Dirac equation for this system is

18 $$(i{\tilde{\gamma }}^{\mu }{\partial }_{\mu })\varphi (x)=0.$$

*ϕ*(*x*), we term this massless mode as Majorana-Weyl fermion mode. This massless mode appears for *μ* = 2*J*. At this point where system shows the topological quantum phase transition through a change of unity of topological number, which corresponds to the appearance Majorana zero modes at both the ends of the array^1, 5^. These gapless edge mode has interesting properties that they are not the same chiral fermion mode that propagate on the edge of integer Hall quantum Hall effect^15^. These are very special because the fermion are chiral and they are also Majorana modes^1^. It can be shown using the Clifford algebra representation, that the condition for the appearance of Majorana-Weyl fermion mode appears for only in the space-time dimension (8*k* + 2), $k=0,1,2\ldots .$ ^1^. Our present problem is (1 + 1) dimension and Majorana-Weyl fermion mode satisfice the condition for (*k* = 0) the appearance in this system. As the Majorana-Weyl fermion mode appears, the system shows the transition from the non-topological state (*W* = 0) to the topological state (*W* = 1).

The massive Majorana fermion mode ($\chi (x)$) and the massless Majorana-Weyl fermion mode (*ϕ*(*x*)) are different. The different wave functions for the topological and non-topological states of the system also satisfy the basic criteria that the topological properties of the system are hidden in the properties of ground state wave function^2^.

Our study shows that the excitations at the topological quantum phase transition is massless and, therefore the system is in the quantum critical state. Hence, the present study reveals that the quantum criticality also exists for the topological quantum phase transition with the emergence of massless Majorana-Weyl fermion mode.

Now we interpret the results from the perspective of interacting light-matter physics. It is clear from our study that mass of the fermionic collective modes is positive for the topological state and negative for the non-topological state. This positive and negative mass of the collective fermionic excitations depend on the difference of *ω*_*p*_ and *ω*_*c*_. When this difference is positive and greater than 2*J*, the collective excitation is like to the Dirac fermion mode, i.e., the system is in non-topological state, otherwise the mass of the collective mode of the system is always positive and the system is in topological state. When the difference between the two frequencies is 2*J* at that point, the collective excitation of the system is the Majorana-Weyl fermion mode.

#### Dirac equation for non-topological state and physical explanation

For the Hamiltonian *H*_2_, in the limit of Δ = 0 and *g* = 0, the Hamiltonian *H*_2_ reduces to

19 $${H}_{3}=-\,J\sum _{j}({\sigma }_{j}^{\dagger }{\sigma }_{j+1}^{-}+h\mathrm{.}c\mathrm{).}$$

After the Jordan-Wigner transformation and the Abelian Bosonization study one can write the above Hamiltonian as^46^

20 $${H}_{3}=\sum _{s}{\int }_{-{\rm{\Lambda }}}^{{\rm{\Lambda }}}\frac{dk}{2\pi }(k{v}_{F})({\psi }_{s,R}^{\dagger }(k){\psi }_{s,R}(k)-{\psi }_{s,L}^{\dagger }(k){\psi }_{s,L}(k))\mathrm{.}$$

Here we use ${\epsilon }_{k}=k{v}_{F}$ near the Fermi points and ${\psi }_{s}^{\dagger }(x)=({\psi }_{s,R}^{\dagger }(x),{\psi }_{s,L}^{\dagger }(x))$. We can write the above Hamiltonian in the following form of Dirac equation without any mass term.

21 $$H=2J\int dx\bar{\psi }(i{\gamma }_{1}){\partial }_{x}\psi $$

and ${\psi }^{\dagger }(x)=({\psi }_{R}^{\dagger },{\psi }_{L}^{\dagger })$, ${\bar{\psi }}^{\dagger }(x)=({\psi }_{L}^{\dagger },{\psi }_{R}^{\dagger })$, ${\psi }_{s}(x)=\int \frac{dk}{2\pi }{\psi }_{s}(k){e}^{ikx}$. It is customary to introduce the Dirac matrices for this equation.

The *γ* matrices are the following. $${\gamma }^{0}=(\begin{array}{cc}0 & 1\\ 1 & 0\end{array})$$, $${\gamma }^{1}=-\,i{\sigma }_{y}=(\begin{array}{cc}0 & -\,1\\ 1 & 0\end{array})$$, $${\gamma }^{5}={\gamma }_{0}{\gamma }_{1}={\sigma }_{z}=(\begin{array}{cc}1 & 0\\ 0 & -1\end{array})$$, where *ψ*_*R*_(*x*) and *ψ*_*L*_(*x*) are the fermionic field for the right and left movers electron. Here, *ψ*_*R*_(*x*) and *ψ*_*L*_(*x*) are the two chiralities each of them is an independent fermionic mode. Although the Dirac equation is massless, still there is no Weyl fermion mode in the system for the following reasons:

The rank of a Dirac spinor depends on the dimensionality. In space-time dimensions *d* = 2*n* and *d* = 2*n* + 1 the Dirac fermion is a complex spinor with 2*n* components (i.e., the form changes every two space-time dimensions). In dimensions d = 4 and higher, it is possible to reduce the Dirac equation to a Weyl equation for two massless spinors of n components. In *d* = 1 + 1, this is not possible and there are no Weyl spinors in 1 + 1 dimensions^14^.

For non-zero values of *g* introduce a term like, ${\psi }_{R}^{\dagger }{\psi }_{R}+{\psi }_{L}^{\dagger }{\psi }_{L}$. This term will only modify the value of *k*_*F*_, but the end result for Dirac equation will be the same. Therefore, the finite values of *g* do not introduce any mass term in the Hamiltonian in the Dirac equation.

Therefore, it reveals from the studies of above two sections that we quantum simulate different relativistic modes for interacting light-matter in a non-linear cavity QED arrays depending on the values of *ω*_*p*_ and *ω*_*c*_.

To the best of our knowledge this is the first attempt for searching the relativistic modes with new and important results in a strongly interacting light-matter physics in a nonlinear cavities array.

### Study of quantized Berry phase with physical explanation

Here, we present the study and results of quantized Berry phase of this model Hamiltonian system. At first, The author of ref. 44 has used the concept of the quantized Berry phase^47^ to define a local topological order parameter for gapped quantum liquid system which do not require any translation symmetry. The author has also considered the anti-unitary symmetric Hamiltonian with the gapped ground state.

Our model Hamiltonian fulfils all the criteria that the author of ref. 44 has proposed to study the quantized Berry phase. There are quite a few studes in the literature of quantum condensed matter physics for searching the topological state and properties of the system through the study of Zak phase^48–63^ but the interacting light matter physics has not been explored yet^30–41^.

To the best of our knowledge this is the first attempt with new and important results for a strongly interacting light-matter physics in a one-dimensional array of nonlinear cavities to study the quantized Berry phase and its relation to the topological quantum phase transition. Berry phase is a geometric phase of eigen state obtained when cyclically varying external parameters. One can write it analytically for the Hamiltonian *H*(*R*) as

22 $${\gamma }_{n}={\int }_{C}\langle n(R)|i{\nabla }_{R}|n(R)\rangle dR.$$

*C* is the closed loop, |*n*(*R*)〉 is the nth eigen vector in the parameter space of *R*.

The topological properties of one-dimensional solids are characterized by the so called Zak phase^49^. Basic definition of Zak phase is the following: The Berry’s phase picked up by a particle moving across the Brillouin zone. Here Brillouin zone is in the one dimension as treated by the Zak, and therefore, the natural choice for the cyclic parameter is the crystal momentum (*k*). The geometric phase in the momentum space is defined as

23 $${\gamma }_{n}={\int }_{-\pi }^{\pi }dk\langle {u}_{n,k}|i{\partial }_{k}|{u}_{n,k}\rangle ,$$

where |*u*_*n*,*k*_〉 is the Bloch states which are the eigen states of the nth band of the Hamiltonian. The ambiguity of the Zak phase problem has been solved by the Atala *et al*.^55^ by considering the difference of Zak phase between the states. This difference of Zak phase between the different states could be a proper topological number^55^. In our present study the difference of Zak phase between the topological state and the non-topological state is *π* and appears when the topological quantum phase transition occurs in the system. Therefore, in the present problem the quantized Zak phase correctly present the topological properties of the system.

The present system is the one dimensional *Z* type topological invariant system, and the system has the anti-unitary particle-hole symmetry to ensure that the curve *C* can only be in a great circle on the Bloch sphere^48^. This Zak phase expresses as $\gamma =\frac{{\rm{\Omega }}(C)}{2}$, where *C* is the close loop that the Hamiltonian forms on the Bloch sphere when *k* varies from −*π* to *π* and ${\rm{\Omega }}(C)$ is the solid angle of the surface enclosed by the curve *C*. Therefore, the Zak phase is either *π* or 0. The analytical relation between the winding number, *W*, with the geometric phase is the following^48^.

24 $$\gamma =W\,\pi \,mod\mathrm{(2}\pi ).$$

We find the values of *W* either 1 or 0. Therefore, the corresponding Zak phase is *π* or 0^42, 48^. The transition of *γ* from *π* to 0 occurs when the system shows the topological quantum phase transition from a topological state to non-topological state.

**Figure 4:**
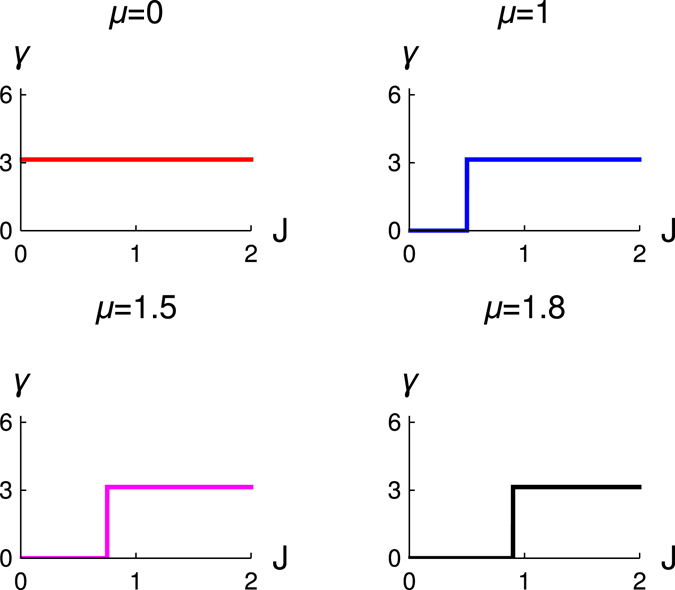
The variation of Zak phase (*γ*) with *J* for different values of *μ*. We study for the four different values of chemical potentials *μ* = 0(*red*), 1(*blue*), 1.5(*magenta*), 1.8(*black*), Δ = 1.

Figure 4 shows the variation of *γ* with *J* for different values of chemical potential. We observe that for *μ* = 0, the geometric phase of the system is always finite (*π*). For the other values of *μ*, we predict that geometric phase shows a sharp transition from the value *π* to 0 for *J* = *μ*/2. The behavior of quantized Zak phase is same for all finite values of Δ (here we consider Δ = 1).

**Figure 5:**
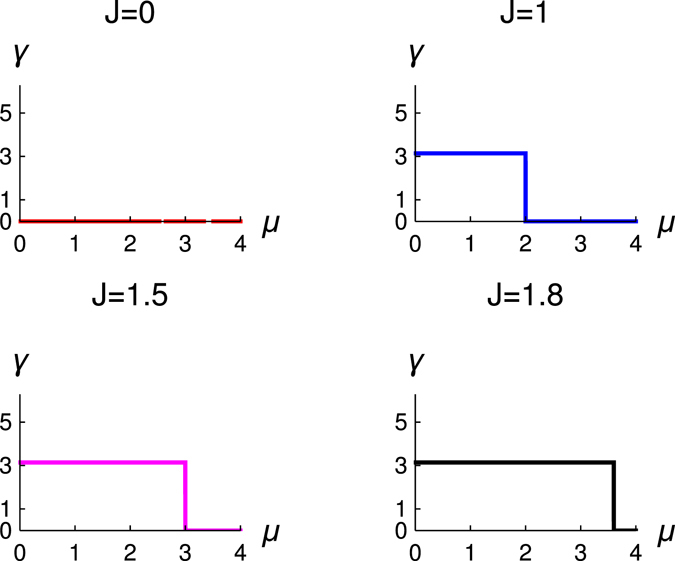
The variation of Zak phase (*γ*) with *μ* for different values of *J*. We study four values of *J* = 0.5, 1, 1.5, 1.8 for the colour red, blue, magenta and black respectively, Δ = 1.

In Fig. 5, we also study the behavior of geometric phase with *μ* for different values of *J*. We observe a transition of geometric phase from *π* to 0 when *μ* = 2*J*. This behavior of quantized Zak phase is consistent with the topological quantum phase transition of the system. We would like to explain this result more physically, the model Hamiltonian parameters Δ and *J* are equal, and also $\mu \ne 0$, i.e., the finite detuning between the cavity frequency and the parametric pumping leads to the topological quantum phase transition. But for the resonance condition, i.e., when *ω*_*p*_ = *ω*_*c*_ system is in the always topological state. Therefore, depending on the difference of the values of *ω*_*p*_ and *ω*_*c*_, system is either in topological state or in non-topological state. Therefore, we conclude that the basic topological properties of the system also find through the study of quantized Zak phase. Hence, we prove in our study the equivalence between the topological number and the local topological order for this system Hamiltonian. This study and results are entirely new in the literature of interacting light matter system^30–41^.

### Experimental proposal and implementation

There are quite a few proposals to detect the Majorana fermion mode in the previous studies^6, 64, 65^. But the authors of ref. 40 have proposed a very elegant process for the detection of Majorana alike fermion modes in optical Kitaev’s chain. The fermionized photons are intrinsically spinless and the pairing occurs between nearest-neighbour cavities only as we discuss in the previous section. The chemical potential (*μ*) can be changed very easily by tuning the resonance frequency of the individual cavities and the driving frequency *ω*_*p*_. The phase and amplitude of the superconducting order parameter can be achieved by regulating the phase and amplitude of the two photon parametric driving. The tunneling amplitude of photons can be regulated by introducing intermediate control device between the cavities. This level of control on the parameters is the key to overcoming difficulties faced by the other solid state systems-based proposals^64, 65^.

This optical detection scheme is simple, versatile and physically realizable but the photon loss from the cavity is unavoidable in this system and this limits the time-scale of the detection; i.e., the time scale should be much shorter than the time scale of photon life time (~$\tfrac{1}{{\rm{\Gamma }}}$, where ${\rm{\Gamma }}$ is the cavity decay), for the observation of Majorana fermion mode.

Now we are interested to discuss the physics of Majorana fermion mode under this dissipation, in the Majorana fermion physics the parity breaking process is the most important one. All the parity breaking dissipation channel can be considered as a single effective parity breaking channel^40^. We already express our model Hamiltonian as an optical Kitaev chain (Eq. 3), the open nature of this chain can be expressed by introducing an effective single-particle loss term in the dynamics^40^.

We try to understand the single particle losses using the Lindblad equation.

25 $${\partial }_{t}\rho =-\,i[{H}_{1},\rho ]+{\rm{\Gamma }}\sum _{i\mathrm{=1}}^{N}({b}_{i}\rho {b}_{i}^{\dagger }-\frac{1}{2}\{{b}_{i}^{\dagger }{b}_{i},\rho \}).$$

where *ρ* is the density matrix of the system, *H*_1_ is the Hamiltonian (Eq. 3) and ${\rm{\Gamma }}$ is the effective single particle decay rate associated with single cavity. ${b}_{i}^{\dagger }({b}_{i})$ is the creation (annihilation) operator of dressed photon for each cavities. One can study the photon losses in the cavity QED lattice from Eq. 25. It reveals from our studies in the previous section that the system is in the topological states for 0 < *μ* < 2*J*. It is well known that for this situation an exponentially localized Majorana fermion mode exists on the both sides of the array with a length scale that increase with *μ* and diverges as *μ* approaches to 2*J* ^5^. For a finite (small) length scale, in the topological state, these Majorana zero modes are weakly coupled and the levels of Majorana qubit that they form split in energy ${\epsilon }_{M}$ (= ${e}^{-\tfrac{L}{\xi }}$, $\xi$ is the localization length of zero energy Majorana fermion mode) which is non-zero and is a small quantity much lesser than the energy gap of the optical Kitaev’s chain (*E*_*g*_) (please see the “Method” section for the detailed analysis).

Now we follow the experimental proposal of the authors of ref. 40 because the model Hamiltonian of the present study is the same as that used in ref. 40. Now we consider two extra nonlinear cavities, one at the left end (*L*) and the other at the right end (*R*). Both of these cavities have set up in the driving frequency *ω*_*p*_ and a tunneling coupling (*J*_*L*_ and *J*_*R*_ are the tunneling coupling for the left and right cavity respectively.) which couple them to the optical Kitaev’s Hamiltonian, *H*_1_.

The low energy effective Hamiltonian in presence of the probe cavities is the following^40^:

$${H}_{eff}={\epsilon }_{M}{\sigma }_{M}^{z}-{J}_{L}{\sigma }_{L}^{x}{\sigma }_{M}^{x}-{J}_{R}{\sigma }_{M}^{x}{\sigma }_{R}^{x}.$$

Where ${\sigma }_{M}^{z}=i{c}_{1}{c}_{2N}={{\rm{\Pi }}}_{j=1}^{N}\,{(-{\sigma }_{j})}^{z}{\sigma }_{1}^{x}{\sigma }_{N}^{x}$ and ${{\rm{\Gamma }}}_{L}$ and ${{\rm{\Gamma }}}_{R}$ are respectively the decay rate of the probe cavity for the left end and right end. *J*_*L*_ and *J*_*R*_ are the photon tunneling amplitude from the left cavity to Kitaev chain and from the right cavity to the Kitaev’s chain respectively. The physical interpretation of the above Hamiltonian is the following. The non-local Majorana qubit is formed by the localized Majorana modes. Majorana modes of the chain mediates a nonlocal coherent exchange of photons between the probe cavities. The authors have shown that under the assumption of the decay rate of probe cavities, i.e., ${{\rm{\Gamma }}}_{L,R}\sim {J}_{L,R}\ll {\epsilon }_{M}$ so that the spontaneous emission occurs on a time scale much larger than the ${t}_{M}(\sim \frac{1}{{\epsilon }_{M}})$ over which correlations are generated. At that point, one finds a direct evidence of Majorana modes in the second order photon cross correlation between the light emitted from the two probe cavities. In numerical simulation of full optical Kitaev’s Hamiltonian coupled to the probe cavities shows signature of nonlocal photon bunching for the Majorana zero mode for small decay rate (${\rm{\Gamma }}\sim {{\rm{\Gamma }}}_{L/R}\ll {\epsilon }_{M}$), and this behavior is observed for small enough system size^40^.

### Implementation

Here we present the implementation of our experimental proposal to detect the Majorana fermion mode. The circuit QED (superconducting circuit based cavity QED) allows for the experimental realization with a sufficient control over dissipation to detect the Majorana fermion modes, i.e., the topological state of the system^66, 67^. As we have understood from our study of the topological properties of optical Kitaev’s chain that the presence of strong interaction between the light and matter and the weak photon loss are the two main ingredients to predict the evidence of topological state in circuit QED lattice. Both the criteria are satisfied in circuit QED. In circuit QED, fabrication and control is in the state of art^66, 67^ and at the same time one can do the quantum state engineering at the desired level.

The cavity QED system consists of a chain of capacitatively coupled identical microwave resonators which play the role of cavities. Each of the cavity has two superconducting transmon qubits which plays the role of artificial atoms^66, 67^. The transmon qubits are placed at an antinode of the intercavity field, the coupling strength between the cavity-qubit is $\lambda \sim {\omega }_{b}\sqrt{\alpha }$, where *ω*_*b*_ is the bare-cavity resonance frequency and *α* is the fine structure constant. The strength of the effective on-site interaction with lower polariton mode is $U=(2-\sqrt{2})\lambda$. The second qubit is placed in between the NN cavities. The external microwave field with a frequency 2*ω*_*p*_ drives the second qubit periodically which creates and annihilate pairs of photons of frequency *ω*_*c*_ = *ω*_*b*_ − *λ*. In the presence of large coupling strength between the cavity and qubit compared to the amplitude of parametric drive which only annihilates and creates photon pairs in the NN cavities. Finally it creates an effective p-wave pairing.

In the circuit QED, one can estimate the parameter space as $\lambda \sim 0.1{\omega }_{b}\sim {10}^{4}\,{\rm{\Gamma }}$, where ${\rm{\Gamma }}$ is the decay rate of cavity photon mode. One can simulate this regime of parameter space through the quantum state engineering and also the inequality condition of parameter space $U\sim \lambda \gg J$, $|{\rm{\Delta }}|\gg {\rm{\Gamma }}$. The first condition of inequality implies that system is in the strong coupling regime, where the photon is fermionized photon which finally leads to the optical version of Kitaev’s chain. If we adjust the detuning field (*μ*) between the cavities and the pump such that |*μ*| < 2*J*. At this quantum state of engineering the system is in the topological state with Majorana fermion mode with an energy splitting ${\epsilon }_{M}$ that depends on *J*, |Δ|, |*μ*| and vanishes exponentially with system size *L* (please see the “Method” section for detail discussion). The second relation of the inequality determines the broadening of Majorana level. In order to exist the topological state, this broadening should be smaller than the energy levels splitting ${\epsilon }_{M}$ and also *E*_*g*_. So that Majorana energy levels do not overlap with energy level of bulk. The system shows topological quantum phase transition (for |*μ*| < 2*J*) and the Majorana fermion modes collective excitation changes to Majorana-Weyl fermion mode excitations at the point |*μ*| = 2*J* and when |*μ*| > 2*J* the collective modes of the system changes to Dirac fermion mode. To do these quantum state engineering, the condition ${\epsilon }_{M}\gg {\rm{\Gamma }}$ is generally more restrictive than ${E}_{g}\gg {\rm{\Gamma }}$. As far as the condition *J*, $|{\rm{\Delta }}|\gg {\rm{\Gamma }}$ is conserved, all these condition satisfy by tuning *μ* well inside the topological state. Therefore, this condition is the sufficient condition for the detection of the topological state of the system up to the tuning of *μ*. But when this inequality is violated, the collective excitation of the system is Dirac like mode.

## Discussions

We have found the topological state and topological quantum phase transition in this system for different regime of parameter space. The topological quantum phase transition occurs only for finite detuning process. We have presented a few results based on the exact solutions along with physical explanation. We have predicted the quantized Zak phase which corresponds to the local topological order. We also prove the equivalence between the topological number and the local topological order from our study. We have also presented the emergence of massive Majorana fermion mode, massless Majorana-Weyl fermion mode and the massless Dirac fermion mode for the different quantum states of this system based on the exact solution. Finally we have presented the experimental proposal and implementation.

## Methods

This section consists of five parts. First, we show explicitly that the phase of the parametric pumping has no effect in the topological properties of the system. In the second, we present the Bogoliubov transformation and the excitation spectrum of the system. In the third one, we present the different representation of winding number calculations and their equivalence to the winding number calculation of Anderson pseudo spin approach^45^. In the fourth one, we present a detailed derivation of emergence of Dirac equation for Majorana fermion mode and massless Dirac equation for non-topological state. Finally, we present, the analytical expression for ${\epsilon }_{M}$ and *E*_*g*_ with necessary discussions.

### Phase independent behavior of Hamiltonian for the topological study

One can also write the above Hamiltonian in the Majorana basis as follows:

26 $$\begin{array}{rcl}H & = & -J\sum _{i=1}^{N-1}({e}^{i\varphi \mathrm{/2}}{a}_{i}+{e}^{-i\varphi \mathrm{/2}}{a}_{i}^{\dagger })({e}^{i\varphi \mathrm{/2}}{a}_{i+1}+{e}^{-i\varphi \mathrm{/2}}{a}_{i+1}^{\dagger })\\  & = & -2iJ\sum _{i\mathrm{=1}}^{N-1}{\gamma }_{i,B}{\gamma }_{i+1,A}.\end{array}$$

${\gamma }_{i,B}=\frac{1}{\sqrt{2}}({e}^{i\varphi \mathrm{/2}}{a}_{i}+{e}^{-i\varphi \mathrm{/2}}{a}_{i}^{\dagger })$, ${\gamma }_{i,A}=\frac{1}{i\sqrt{2}}({e}^{i\varphi \mathrm{/2}}{a}_{i}-{e}^{-i\varphi \mathrm{/2}}{a}_{i}^{\dagger })$, *γ*_*i*,*B*_ and *γ*_*i*,*A*_ are the Majorana operators. Therefore, it is clear from the above Hamiltonian that in the basis of Majorana fermion the phase *ϕ* has no effect in the topological state of the system.

### Bogoliubov transformation and quasiparticle spectrum

One can diagonalize the Hamiltonian by using the Bogoliubov transformation.

The Bogoliubov quasi-particle operators is ${\beta }_{k}=\,\cos \,({\theta }_{k}\mathrm{/2)}\,{c}_{k}+i\,\sin \,({\theta }_{k}\mathrm{/2)}\,{c}_{-k}^{\dagger }$.

The analytical expression for cos *θ*_*k*_ and sin *θ*_*k*_ are $\cos \,({\theta }_{k})=\frac{-\,2J\,\cos \,k-\mu }{{E}_{k}}$, and $\sin \,({\theta }_{k})=\frac{2{\rm{\Delta }}\,\sin \,k}{{E}_{k}}$ respectively, where ${E}_{k}=\sqrt{{(\mu +2J\cos k)}^{2}+4{{\rm{\Delta }}}^{2}\,{\sin }^{2}\,k}$. The Bogoliubov quasiparticle operator diagonalize the Hamiltonian. Finally, the Hamiltonian reduces to $H(k)={\sum }_{k}\,{E}_{k}{\beta }_{k}^{\dagger }{\beta }_{k}$.

### Different representation of winding number and their equivalence

One can find the analytical expression for winding number calculations by the following analytical expression (Eq. 27). Finally we prove that this analytical expression is the same as that of winding number calculation of Anderson pseudo-spin approach^45^.

The effective Hamiltonian of the system is $H(k)=\vec{\chi }(k\mathrm{).}\vec{\tau }$. The other representation of topological invariant for $\chi (k)$ is then expressed by ref. 29.

27 $${W}_{2}=\frac{1}{4\pi }\int {\epsilon }_{\alpha \beta }\frac{1}{{\hat{\chi }}_{\alpha }}\frac{\partial {\hat{\chi }}_{\beta }}{\partial k}dk.$$

Here *α* and *β* are *y* and *z* two components and ${\epsilon }_{\alpha \beta }$ is the antisymmetric tensor. For the present problem, ${\chi }_{x}(k)=0$, ${\chi }_{y}(k)=\,\sin \,{\theta }_{k}=2{\rm{\Delta }}\,\sin \,k$, ${\chi }_{z}(k)=\,\cos \,{\theta }_{k}=-\,2J\,\cos \,k-\mu$.

One can write, Eq. 26, in the following from: ${W}_{2}={W}_{2}^{A}-{W}_{2}^{B}$. ${W}_{2}^{A}={\int }_{-\pi }^{\pi }\,\frac{dk}{4\pi }(\frac{1}{{\chi }_{y}}\frac{\delta {\chi }_{z}}{\delta k})$, ${W}_{2}^{B}={\int }_{-\pi }^{\pi }\,\frac{dk}{4\pi }(\frac{1}{{\chi }_{z}}\frac{\delta {\chi }_{y}}{\delta k})$. Finally we get,

28 $${W}_{2}^{A}=-\,{W}_{2}^{B}=\frac{1}{4\pi }{\int }_{-\pi }^{\pi }\frac{2{\rm{\Delta }}\mathrm{(2}J+\mu \,\cos \,k)}{{(\mu +2J\cos k)}^{2}+4{{\rm{\Delta }}}^{2}\,{\sin }^{2}\,k}dk$$

29 $${W}_{2}={W}_{2}^{A}-{W}_{2}^{B}=\frac{1}{2\pi }{\int }_{-\pi }^{\pi }\frac{2{\rm{\Delta }}\mathrm{(2}J+\mu \,\cos \,k)}{{(\mu +2J\cos k)}^{2}+4{{\rm{\Delta }}}^{2}\,{\sin }^{2}\,k}dk$$

This analytical expression for *W*_2_ is the same as *W* (Eq. 9). One can also prove the equivalence in the following way: We use the analytical expression ${\chi }_{y}(k)$ and ${\chi }_{z}(k)$ and use Eq. 26, and we then finally end up with the Eq. 9.

30 $${W}_{2}={\int }_{-\pi }^{\pi }\frac{dk}{4\pi }(\frac{1}{\cos \,{\theta }_{k}}\,\cos \,{\theta }_{k}+\frac{1}{\sin \,{\theta }_{k}}\,\sin \,{\theta }_{k})\frac{d{\theta }_{k}}{dk}=(\frac{1}{2\pi }){\int }_{-\pi }^{\pi }(\frac{d{\theta }_{k}}{dk})dk.$$

The above equation of *W*_2_ is the same as *W*. Therefore, we prove this equivalence between the different representation of winding number.

### Derivation Dirac equation for massive Majorana fermion mode

Our starting Hamiltonian is *H*_2_ (Eq. 14). We have found from the exact solution that for Δ = *J* > 0 and *μ* = 0, system is in the topological state, and we have also found from the study of winding number, that this topological state continue for *μ* < 2*J*.

In the limit of topological state Δ = *J* and *g* = 0. The Hamiltonian *H*_2_ reduces to in a rotated spin basis as,

31 $${H}_{2}=-\,J\sum _{j}[g{\sigma }^{x}+{\sigma }_{j}^{z}{\sigma }_{j+1}^{z}].$$

Here we define the Dirac spinor, ${\chi }_{1}(n)={\sigma }_{z}(n){\mu }_{z}(n+\mathrm{1/2)}$ and ${\chi }_{2}(n)={\sigma }_{z}(n){\mu }_{z}(n-\mathrm{1/2)}$.

These two fields, ${\chi }_{1}(n)$ and ${\chi }_{2}(n)$ satisfy the following relations, $\{{\chi }_{1}(n1),{\chi }_{2}(n2)\}=2{\delta }_{n\mathrm{1,}n2}$. At the same time, ${\chi }_{1,2}^{\dagger }(n)={\chi }_{\mathrm{1,2}}(n)$ which can be shown very easily by using the relation between order and disorder operator which are given below.

The equation of motion for the *σ*_*z*_(*n*) is the following:

32 $$\frac{\partial {\sigma }_{z}(n)}{\partial \tau }=[{H}_{2},{\sigma }_{z}(n)]={\sigma }_{x}(n){\sigma }_{z}(n)$$

The equation of motion for *μ*_*z*_(*n* + 1/2) is the following:

33 $$\begin{array}{rcl}\frac{\partial {\mu }_{z}(n+\mathrm{1/2)}}{\partial \tau } & = & g{\mu }_{x}(n+\mathrm{1/2)}{\mu }_{z}(n+\mathrm{1/2)}\\  & = & g{\sigma }_{z}(n){\sigma }_{z}(n+\mathrm{1/2)}{\mu }_{z}(n+\mathrm{1/2}).\end{array}$$

Now we use the properties of the *σ* and *μ* operators to derive the equation of motion for the Majorana fields ${\chi }_{1}(n)$ and ${\chi }_{2}(n)$.

34 $$\begin{array}{rcl}\frac{\partial {\chi }_{1}(n)}{d\tau } & = & -{\sigma }_{z}(n){\mu }_{z}(n-\mathrm{1/2)}{\mu }_{z}(n+\mathrm{1/2)}{\mu }_{z}(n+\mathrm{1/2)}\\  & = & +\,g{\sigma }_{z}(n){\sigma }_{z}(n){\sigma }_{z}(n+\mathrm{1)}{\mu }_{z}(n+\mathrm{1/2).}\end{array}$$

35 $$\frac{\partial {\chi }_{1}(n)}{d\tau }=-\,{\chi }_{2}(n)+g{\chi }_{2}(n+1).$$

Similarly the equations of motion for ${\chi }_{2}(n)$ are

36 $$\begin{array}{rcl}\frac{\partial {\chi }_{2}(n)}{d\tau } & = & {\mu }_{z}(n-\mathrm{1/2)}{\mu }_{z}(n+\mathrm{1/2)}{\sigma }_{z}(n){\mu }_{z}(n-\mathrm{1/2)}\\  &  & +\,g{\sigma }_{z}(n-\mathrm{1)}{\mu }_{z}(n-\mathrm{1/2).}\end{array}$$

After a little bit of calculations and using the relation between the disorder operators, we finally arrive the equation of motion of ${\chi }_{2}(n)$ as,

37 $$\frac{\partial {\chi }_{2}(n)}{d\tau }=-\,{\chi }_{1}(n)+g{\chi }_{1}(n-\mathrm{1).}$$

Now we restore the lattice for that purpose, by doing the following transformation: $(r\pm \mathrm{1)}\to (r\pm \alpha )$. Finally one can write the eqs (34) and (36) as

38 $$\frac{\partial {\chi }_{1}(r)}{dt}=-\,\mathrm{(1}-g){\chi }_{2}(r)+g\alpha \frac{\partial {\chi }_{2}}{\partial r}.$$

39 $$\frac{\partial {\chi }_{2}(r)}{dt}=-\,\mathrm{(1}-g){\chi }_{1}(r)-g\alpha \frac{\partial {\chi }_{1}}{\partial r}\mathrm{.}$$

Finally, we write it in a compact form, $({\gamma }^{0}\frac{\partial }{\partial t}+{\gamma }^{3}\frac{\partial }{\partial r}+m)\chi (x)=0$. where ${\chi }^{\dagger }=({\chi }_{1},{\chi }_{2})$ and $m=\frac{1-g}{\alpha }$, $${\gamma }^{0}=(\begin{array}{cc}0 & 1\\ 1 & 0\end{array})$$, $${\gamma }^{3}=(\begin{array}{cc}1 & 0\\ 0 & -1\end{array})$$.

We use the following relation between the order (*σ* operators) and disorder (*μ* operators) during the derivation of Dirac equation of massive Majorana fermion mode^68^.

${\mu }_{z}^{2}=1={\mu }_{x}^{2}$, ${\mu }_{z}(n-\mathrm{1/2)}{\mu }_{z}(n+\mathrm{1/2)}={\sigma }_{x}(n)$. ${\mu }_{x}(n+\mathrm{1/2)}={\sigma }_{z}(n){\sigma }_{z}(n+\mathrm{1)}$, ${\mu }_{z}(n+\mathrm{1/2)}={{\rm{\Pi }}}_{j=1}^{n}\,{\sigma }_{x}(j)$. ${\sigma }_{z}(n)={{\rm{\Pi }}}_{j=0}^{n-1}\,{\mu }_{x}(j+\mathrm{1/2)}$, $[{\mu }_{x}(n+\mathrm{1/2)},{\mu }_{z}(n^{\prime} +\mathrm{1/2)]}=2{\delta }_{n,n^{\prime} }$ $[{\mu }_{z}(n+\mathrm{1/2)},{\mu }_{z}(n^{\prime} +\mathrm{1/2)]}=0$,$[{\mu }_{z}(n+\mathrm{1/2)},$ ${\sigma }_{x}(n^{\prime} )]=0$. It is clear from the above analytical expression of *μ*_*z*_(*n* + 1/2) that it is a kink operator, and it introduces the disorder in the system.

Now, we present the massless Majorana-Weyl equation as

40 $$({\gamma }^{0}\frac{\partial }{\partial t}+{\gamma }^{3}\frac{\partial }{\partial r})\varphi (x)=0,$$

where *ϕ*(*x*) is the solution for massless Majorana-Weyl fermion. The important point to be noted that Weyl fermion occurs at *m* = 0 = 1 − *g* that implies the *μ* = 2*J*, where the topological quantum phase transition occurs.

#### Analytical expression for ${\epsilon }_{{M}}$ and *E*_*g*_ and necessary discussions

Here we follow the seminal paper of Kitaev’s^5^. The energy splitting of the Majorana qubit is ${\epsilon }_{M}\sim {e}^{\frac{-L}{\xi }}$, where *L* is the length of the system and $\xi$ is the localization length of the Majorana zero modes. The analytical relation of *ξ* ^−1^ with the parameters of optical Kitaev’s chain Hamiltonian is

41 $${\xi }^{-1}=min\,\{|ln|{x}_{+}||,|ln|{x}_{-}||\},$$

where ${x}_{\pm }=\frac{-\mu \pm \sqrt{{\mu }^{2}-4{J}^{2}+4{|{\rm{\Delta }}|}^{2}}}{2(J+|{\rm{\Delta }}|)}$. It is very clear from the analytical expression of ${\epsilon }_{M}$ that it vanishes for large values of *L*, i.e., there is no finite overlap between the zero energy nonlocal Majorana fermion modes as a consequences the system is in topological state. But ${\epsilon }_{M}$ is finite when $\xi \to \infty$ and *L* is finite and small.

From the analysis of *ξ* ^−1^ and from *x*_+_ and *x*_−_, we obtain the following relations in our study.

1. *E*_*g*_ = 2*J* − *μ* if Δ ≥ *J* or if Δ < *J* and $2J-\mu  < \frac{2{{\rm{\Delta }}}^{2}}{J}$. *E*_*g*_ is the energy gap of the optical Kitaev’s chain.
2. $${E}_{g}={\rm{\Delta }}\mathrm{(4}-{\mu }^{2})/\sqrt{{J}^{2}-{{\rm{\Delta }}}^{2}}$$ otherwise.
